# Validity Evidence Based on the Content of the MAPS-B Cognitive Assessment Instrument

**DOI:** 10.1192/j.eurpsy.2025.1842

**Published:** 2025-08-26

**Authors:** J. Martini, M. B. Martins, M. R. Zibetti, J. J. Schneider, C. M. Trentini

**Affiliations:** 1 Assessment and Measurement in Psychology, Federal University of Rio Grande do Sul, Porto Alegre; 2 Psychopathological States and Psychotherapeutic Approaches, University of Vale do Rio dos Sinos, São Leopoldo, Brazil

## Abstract

**Introduction:**

The MAPS-B is part of a project focused on the construction of instruments for cognitive assessment in the Brazilian population. Comprising eight subtests, MAPS-B aims to enhance understanding of cognitive functioning in individuals over 50 years old. The subtests assess autopsychic and allopsychic orientation, perception, naming, memory, praxis, visual and auditory focused attention, working memory, automatic language, inhibition, and semantic memory. Validity evidence is essential to ensure the safe use of new instruments in psychological assessments. Content-based validity evidence reflects the degree to which the instrument aligns with and adequately measures the construct of interest. Such evidence can be derived from expert judgment.

**Objectives:**

To investigate the content-based validity evidence of the MAPS-B.

**Methods:**

The analysis involved four judges with expertise in neuropsychological assessment, who completed a questionnaire on MAPS-B’s subtests. The judges assessed the adequacy and clarity of the instructions and the relevance of each subtest for measuring the proposed construct, using a Likert scale (0 to 4). Space was also provided for comments and suggestions. Responses were analyzed using the Content Validity Index (CVI), calculated individually (CVI-I) and globally (CVI-T) for the instrument. A CVI score above 1 was considered acceptable (Yusoff, Education in Medicine Journal Int 2019; 51). Additionally, items with suggestions from the judges were qualitatively reviewed.

**Results:**

Expert analysis indicated total agreement across all subtests, with individual and total CVI scores of 1, demonstrating adequacy in terms of relevance, clarity, and overall suitability. However, qualitative adjustments were suggested for two subtests assessing orientation and perception. Following analysis and consensus among the authors, modifications were made as shown in **Table 1**.

Table 1

**Table:** 

Original Item	Post-Judge Analysis Adjustment
“What part of the day is it?”	A note was added to the manual, allowing assessors to clarify the question by adding, ‘What part of the day are we in? Morning, afternoon, or evening?’”
“What is your address?”	A note was added to the manual specifying that correct answers may include just the street name and house number.
“Mentally assemble it and name it.”	Rephrased to “Can you mentally assemble the figure and name it?”

**Conclusions:**

This study provides content validity evidence for the MAPS-B, showing that its subtests adequately represent the constructs being assessed. All items achieved satisfactory CVI scores in line with literature recommendations (CVI > 1), indicating agreement on item relevance and suitability. The qualitative suggestions from experts contributed to refining the MAPS-B. Although the current results are satisfactory for the instrument’s proposed use, future studies are needed to gather further validity evidence and investigate the instrument’s reliability.

**Disclosure of Interest:**

None Declared

